# Advancing hydrology for societal impact: integrating transdisciplinary frameworks to bridge research and practice

**DOI:** 10.1098/rsta.2024.0283

**Published:** 2025-07-31

**Authors:** Caitlyn Hall, Jacqueline Melvold

**Affiliations:** ^1^ College of Agriculture, Life, and Environmental Sciences and College of Engineering, The University of Arizona, Tucson, AZ, USA; ^2^ Transdisciplinary (TD) School, University of Technology Sydney, Sydney, New South Wales, Australia; ^3^ W.A. Franke Honors College, The University of Arizona, Tucson, AZ, USA

**Keywords:** transdisciplinary, climate resilience and adaptation, hydrology, knowledge translation, co-production

## Abstract

As hydrology faces the mounting pressures of climate change, researchers grapple with translating their technical advancements into societal impact. Traditional hydrology research often isolates technical solutions, such as optimizing water flows or predicting droughts, from the broader socio-economic, ecological and policy contexts in which water issues unfold. This compartmentalization limits the effectiveness of hydrological research in addressing complex, interconnected water challenges. Transdisciplinary (TD) approaches offer a path forward, emphasizing the integration of diverse knowledge sources, the co-production of solutions with community and policy stakeholders and a reflexive stance that acknowledges researchers’ positionality and assumptions. This article advocates for adopting well-established frameworks from other fields to transcend disciplinary boundaries, fostering actionable solutions that resonate with societal needs. By leveraging structured frameworks to manage technical uncertainties, synthesize knowledge and prioritize contextual relevance, hydrologists can enhance the real-world impact of their research. We propose four guiding principles for incorporating TD practice into hydrology and offer five guiding questions to support hydrologists in guiding their shift towards societal impact. We aim to build a research practice that advances scientific understanding and addresses urgent water challenges through collaborative, socially engaged and context-sensitive approaches.

This article is part of the Royal Society Science+ meeting issue ‘Hydrology in the 21st century: challenges in science, to policy and practice’.

## 1. Advancing hydrology research to practice for societal impact

As societal questions and challenges around water become increasingly complex, many hydrology researchers have shared visions of the most pressing problems and opportunities within hydrology. However, these advances must often be revised when applied to climate change’s complex and interconnected challenges. One of the critical constraints is that traditional hydrology research tends to focus on narrowly defined problems, such as optimizing water flows, predicting drought or enhancing groundwater recharge, while neglecting broader social, ecological and economic dimensions [1]. Blöschl *et al*. [1] identified that 20 of the 23 most critical unsolved problems in hydrology were to address technical questions. The authors identified primary barriers to overcome: understanding phenomena, improving measurements and developing technical methods [1]. Though valuable, isolated technical solutions often struggle to address the full spectrum of real-world complexities. While Blöschl *et al*. [1] acknowledge hydrology as a practice-linked, cross-cutting discipline, only three unsolved challenges at the hydrology–society intersection focus on its impact on people: improving communication of uncertainties, understanding synergies and trade-offs in water management and exploring water’s role in societal dynamics to guide modern water management. While many in the community are working to address challenges in knowledge exchange and translating hydrology research to actionable solutions and change, there is still a broad underemphasis on taking research beyond academia.

Rokaya & Pietroniro [2] argue that while hydrology has made significant technical strides, it has often fallen short of adequately addressing the broader, interconnected systems in which water challenges exist. While variability can significantly influence the transferability of findings from local studies to regional or global contexts, experimental methods often fail to account for the rapid changes induced by climate variability and extreme events, thus limiting their applicability in forecasting or managing long-term water availability [3]. Many have highlighted the importance of moving beyond isolated technical solutions to embrace a systems-thinking approach that considers the feedback loops between water systems, human activity and climate dynamics [4,5]. Optimizing water flows or predicting drought may solve immediate issues, but neglecting socio-economic drivers of water demand or ecological impacts can lead to new or worsened problems.

Moreover, neglecting to fully engage interested parties and actors (e.g. local communities, practitioners and policymakers) in the co-creation of knowledge and solutions means that hydrology research often lacks the contextual relevance needed for practical implementation [6–8]. This gap between research and practice leads to a disconnect where scientific findings remain underutilized in water-related practices and policies [2,9]. Addressing these limitations requires rethinking traditional hydrology to incorporate more integrative, transdisciplinary (TD) approaches that emphasize the importance of engaging interested parties, knowledge translation and the co-production of actionable solutions that align with technical realities and societal needs for transformative action [10,11].

## 2. Transdisciplinarity as a tool for hydrology

Arheimer *et al*. [6] emphasize the importance of moving beyond traditional hydrology approaches, calling for ‘transdisciplinary evidence to ensure actionable and transferable solutions to water threats around the globe’. Many researchers are increasingly turning to TD approaches to tackle context-specific water challenges (e.g. [12–14] and to conduct meta-evaluations of how we conduct hydrology research (e.g. [15–17 ]). Focusing on impact-driven research through TD methods can drive interdisciplinary thinking by integrating ideas from diverse contributors, spanning disciplines and knowledge sources [18].

TD emerged to move science and education beyond interdisciplinary boundaries, promoting broad innovation rather than confining research within disciplinary and sociocultural (19,20). TD encourages researchers to challenge these silos, fostering a comprehensive understanding of complex systems. By engaging practitioners, communities and diverse knowledge holders—including local and indigenous knowledge—TD facilitates the development of actionable, context-specific solutions [21,22]. It also encourages the development of new theories and methods through collaborative, creative thinking that transcends academia for innovative solutions to complex problems. TD can be beneficial for addressing hydrological challenges, like scarcity, pollution control, climate adaptation and public health concerns related to clean water access, sanitation and hazard resilience. This approach also supports hydrology-related energy transitions, such as hydropower sustainability, integrated water-energy planning and social and governance issues like equitable water allocation and transboundary water conflict resolution. In agriculture and food security, TD methods enable solutions for sustainable irrigation practices and mitigating the hydrological impacts of climate variability. TD approaches reveal hidden assumptions within disciplines and create a space for mutual learning, fostering contextual awareness and prioritizing human benefit and societal improvement [23,24].

Many researchers may feel the need to create TD approaches, but we propose that hydrologists leverage existing TD frameworks to develop and translate research. By examining established TD frameworks from other fields, researchers can avoid ‘reinventing the wheel’ and focus on creating solutions. Established TD frameworks, such as joint problem framing, can lower barriers to adopting novel approaches—especially for early career scientists and those driving urgent, climate-responsive action ([25–27]) . Herein, we provide examples and guidance on incorporating TD approaches to view problems and solutions through diverse lenses, advancing hydrology research.

## 3. Exploring TD frameworks to drive effective hydrology research

We present three TD frameworks to advance hydrological research and translate knowledge into real-world solutions. These frameworks aim to drive tangible, lasting change by integrating diverse knowledge systems, fostering interdisciplinary collaboration and engaging stakeholders to co-produce actionable solutions. Focusing on co-design, problem framing, knowledge synthesis and implementation, they address the multifaceted nature of hydrological systems, balancing ecological, social and technical considerations. This integrated approach has effectively addressed water-related issues across diverse contexts, such as urban water management in Singapore, sustainable groundwater practices in India’s Punjab region and transboundary water governance in the Nile Basin. These frameworks enable hydrology researchers to generate scientifically rigorous and socially relevant solutions, fostering innovation while addressing pressing challenges such as water scarcity, flooding and climate adaptation. These frameworks ensure that hydrology research translates into sustainable and equitable outcomes by prioritizing inclusivity, scalability and practical application.

While many TD frameworks exist, not all are well-suited for hydrology research due to water-related challenges, unique socio-environmental dynamics and scale. For example, frameworks like Mode 2 knowledge production, which focus primarily on applied, problem-driven research, may lack the structured mechanisms for stakeholder engagement and practical implementation required in hydrology. The lack of structured co-production has often led to fragmented or uncoordinated approaches because of its limited success in transboundary water governance. Similarly, the post-normal science framework, while emphasizing the importance of uncertainty and stakeholder inclusion, tends to prioritize debates over solutions. This framework approach may stall progress in time-sensitive issues such as drought management in Sub-Saharan Africa. Additionally, strong community involvement frameworks like participatory action research may fall short in integrating scientific data and technical rigour, limiting their applicability to challenges like groundwater modelling or urban flood mitigation. In contrast, the Ten Essentials, Integration and Implementation Sciences (i2S) framework and ISOE model integrate diverse perspectives and emphasize co-creation, synthesis and actionable outcomes, making them relevant for hydrology research. We explore how these frameworks can be applied to hydrology research, helping to overcome disciplinary barriers, integrate diverse perspectives and develop actionable solutions that address critical questions.

### (a) The Ten Essentials for TD research

In the first framework, the Ten Essentials framework, Fazey, 2019 [28] offers a reflexive and positionality-oriented approach to TD research to achieve a greater focus on action and solution-oriented outcomes for complex, urgent issues. This framework emphasizes the importance of transformative solutions, practical knowledge and the integration of diverse perspectives to foster systemic change [28]. This framework outlines ten essential steps that encourage researchers to adopt a solution-driven, holistic and reflexive approach, urging them to move beyond incremental improvements to address structural challenges embedded in societal and environmental systems. This framework emphasizes recognizing that researchers are inextricably linked to their work, which is inherently shaped by their positionality and the sociocultural context of their research.

This reflexive and context-sensitive approach has significant implications for hydrology, where researchers increasingly acknowledge the importance of positionality and sociocultural context in shaping water-related knowledge and translating it into actionable solutions. For instance, geoscientists explore how their backgrounds, values and local contexts impact their research focus and approach (e.g. [16,29,30]). Work by hydrologists has highlighted the importance of reflexivity and ethical awareness in water research, emphasizing that researchers have the power to meaningfully engage with the communities affected by water challenges to co-create robust and socially relevant science. By applying the Ten Essentials framework, hydrologists can embrace a more context-sensitive approach to help align their research to address the complex realities of water management in a changing climate.

### (b) The Integration and Implementation Sciences (i2S) framework

The second framework, the i2S framework, developed by Bammer, 2019 [31], offers a structured approach for conducting TD research by focusing on three core domains: synthesizing knowledge across disciplines, managing unknowns and supporting the implementation of research for practical use. This framework includes ‘gut check’ questions that enable researchers to critically evaluate their aims, existing and needed knowledge, uncertainties, methods and the potential for integrating findings into decision-making. By framing problems in terms of knowns and unknowns, i2S provides a systematic way to identify limitations and assess the applicability of research findings in real-world contexts.

The i2S framework’s focus on synthesis and decision support is highly relevant to hydrology, where researchers face the challenge of translating complex water-related findings into actionable insights [32]. For instance, when addressing uncertainties in water scarcity projections or flood risk assessments, hydrologists can apply i2S principles to critically evaluate and synthesize diverse sources of knowledge—from climate models to local observations—into cohesive strategies for action. In Singapore, Rahman *et al*. [33] used the i2S framework to integrate engineering, policy and public engagement for sustainable urban water solutions, while in Bangladesh, stakeholder engagement and knowledge synthesis were central to co-developing flood risk mitigation strategies in vulnerable communities. Similarly, in the Aral Sea Basin, i2S principles guided efforts to tackle transboundary water governance issues and promote sustainable water allocation among Central Asian countries [34]. This approach aligns with the guiding principles outlined by Rangecroft *et al*. [30], who emphasize the importance of ethical considerations, effective communication and adaptable research practices when working with diverse stakeholders. By applying the i2S framework, hydrologists can bridge disciplinary gaps and address scientific and societal needs such that research outcomes are robust and relevant to decision-makers and community needs.

### (c) The ISOE model for TD research

The third framework, the ISOE model developed by Bergmann and Jahn, 2017 [35], provides a structured approach to TD research by integrating problem framing, knowledge synthesis and societal participation throughout the research process. This model emphasizes the importance of co-producing knowledge with diverse societal actors, such that research addresses real-world challenges while remaining scientifically robust and contextually relevant. Researchers also helped local communities in Namibia combat water scarcity by developing innovative rainwater harvesting and wastewater treatment systems through TD research with ISOE team members [36]. Similarly, in the Middle East, an approach similar to the ISOE model addressed water–energy–food interdependencies, promoting sustainable resource management in arid regions [37]. By involving key interested parties from the outset, this model supports the development of research that reflects the practical needs and perspectives of those most affected by the research problem because it requires the re-integration of findings with societal relevance.

The ISOE model’s emphasis on participatory problem framing and continuous integration of diverse knowledge systems is particularly relevant in hydrology, where complex water-related issues require nuanced, localized solutions. For instance, in addressing water scarcity, pollution or flood risk management, hydrology researchers can apply this model to engage communities, policymakers and industry actors to facilitate implementable research outcomes aligned with local needs and values. Rangecroft *et al*. [30] exemplify this approach in hydrology by outlining principles that respond to communication, ethical considerations and the practicalities of working with diverse stakeholders. Hydrologists can co-develop context-sensitive solutions that bridge the gap between scientific understanding and societal action through this model.

### (d) TD framework comparison

Each framework offers distinct advantages in advancing hydrological research. Table 1 lists a summary of their key focus areas, along with the strengths and weaknesses of each approach in hydrological research. The Ten Essentials for TD research provides hydrologists with a reflective approach, making it highly effective for creating socially relevant, ethically conscious research. Its emphasis on positionality and context aids in developing water solutions that consider local values, bridging scientific rigour and community needs. On the other hand, the i2S framework is particularly beneficial for integrating diverse knowledge sources, making it ideal for synthesizing complex hydrological data, such as those needed for effective water resource management. This framework also supports decision-making by systematically addressing uncertainties, making it valuable for hydrology’s predictive aspects. The ISOE model fosters collaborative relationships with communities and stakeholders, aligns hydrological research with local challenges and supports the implementation of findings. This model’s emphasis on engagement helps researchers create solutions tailored to specific hydrological issues, such as pollution or flood risk.

**Table 1.** Summaries of the three TD frameworks and their strengths and weaknesses for hydrology research and practice.

framework	key focus	strengths in hydrology research and practice	weaknesses in hydrology research and practice
Ten Essentials for TD research	reflexive and positional approaches that support transformative, action-oriented outcomes in tackling complex problems	— highlights how researchers’ positionality and context shape outcomes, ensuring a relevant and ethical approach — build deep engagement with communities, linking scientific rigour with social relevance to address critical water issues	— reflective and positional analysis can require significant time and commitment — may be challenging for those newer to reflexive practice, demanding skill development in self-awareness and critical reflection
Integration and Implementation Sciences (i2S) framework	systematic approach to synthesize knowledge, manage uncertainties and support decision-making across disciplines	— offers a structured, methodical approach for integrating diverse knowledge sources to handle complex hydrological issues — provides a valuable lens for managing uncertainties in areas like water scarcity and flood risk, supporting action-oriented insights	— high levels of coordination and resources required for managing uncertainties can strain smaller teams — the cross-disciplinary demands are challenging without well-aligned resources and collaborative networks
ISOE model for TD research	co-produced knowledge through participatory problem framing with diverse societal actors	— emphasizes early and consistent engagement with communities, industry and policymakers, aligning hydrology research with broader societal needs — ideal for local and context-driven water challenges like pollution control and water scarcity, grounding research in actionable outcomes	— requires significant resource investment to maintain stakeholder engagement over time — stakeholder fatigue may occur without careful balance in engagement, especially with large and diverse groups

Despite their strengths, each framework also presents unique challenges for hydrology. The Ten Essentials framework’s intensive focus on reflexivity and positionality can be time-consuming and may demand skill development in self-awareness, making it challenging for those less experienced in reflexive practices. The i2S framework requires significant coordination and resources to integrate knowledge and manage uncertainties, which may be difficult for smaller teams or research groups lacking extensive networks. In addition, the ISOE model faces potential limitations due to its high demands for sustained stakeholder engagement, which requires ongoing resources and may lead to collaborator fatigue. This challenge is particularly relevant in hydrology when engaging large or diverse groups over time. While each framework contributes valuable tools for hydrology, researchers could select a model or elements of a model that aligns with their project’s goals, resources and the desired level of stakeholder collaboration.

## 4. TD principles for societally effective hydrology research

The frameworks presented offer some guidance for conducting societally effective research. However, effective research does not always require strict adherence to a particular framework. Instead, researchers can draw inspiration from these models to identify principles that align with their goals, available resources and desired level of stakeholder engagement. Drawing on the strengths of the presented frameworks, we propose four key principles as starting points for hydrologists seeking to enhance the societal impact of their work. These principles are: (i) translating and applying knowledge to develop transformative solutions; (ii) incorporating diverse knowledge systems and co-producing research; (iii) integrating varied perspectives to foster adaptive water research; and (iv) promoting reflexivity and flexibility throughout the research process. Below, we explore each principle in detail, providing examples of how hydrologists can implement these ideas to create meaningful societal change.

These strategies represent a substantial shift for hydrology, as researchers must navigate systemic barriers that have traditionally limited the potential practical application of their work. Challenges such as limited funding, outdated evaluation metrics and institutional preferences for traditional research outputs can hinder progress [38–40]. While individual researchers alone may lack the power to enact these changes institutionally, there is growing support among international bodies for TD research approaches that emphasize real-world impact. For example, the OECD has recognized the importance of TD approaches and issued guidelines encouraging funding agencies to expand the definition of ‘scientific excellence’ to include research with measurable societal, policy and practice impacts [41]. This institutional recognition signals a positive shift towards valuing research that supports transformative solutions, helping pave the way for hydrology to contribute more directly to global water resilience.

### (a) Emphasizing knowledge translation for transformative solutions

To address today’s water challenges, hydrology research must go beyond incremental scientific advancements and shift towards effective knowledge application to drive change [32]. Prioritization of developing accessible knowledge for translation and advancement applies to foundational and applied research, as both are critical to driving transformative change. Hydrology researchers aiming to facilitate the application of their work to address pressing water-related challenges may benefit from considering the sociocultural, political and economic systemic structures that influence water management practices, priorities and policies. This perspective can help drive the goal that research-driven solutions align with the broader context and needs of the communities they serve.

Effective knowledge translation at all research scales, from foundational to applied, is essential because each level of research builds on the other, creating a continuum of insights that drive progress and innovation. Observational hydrology, for example, uses remote sensing and on-the-ground data collection to monitor variables such as hydrological cycles, snowpack dynamics and water quality [42]. While these methods provide invaluable insights, challenges remain in translating data into actionable information for decision-makers. For instance, data often lack the temporal resolution to capture sudden events like flash floods or prolonged droughts [43]. Furthermore, without clear contextualization—answering ‘What does this data mean?’—decision-makers may struggle to use it effectively for policy or hazard mitigation. Although powerful, advanced instruments and analytical tools can be prohibitively costly or inaccessible, particularly in resource-limited settings, limiting the scalability and societal impact of hydrological research. This gap underscores the need for approaches that make data more actionable and accessible to support practical water management decisions. Hydrologists can explore this principle by considering the following:

— *Target root causes of water challenges.* Hydrology researchers should consider how their research addresses foundational drivers of water challenges versus immediate issues. For example, when exploring challenges related to water scarcity, hydrologists can explore how their work relates to and can impact the fundamental causes of water scarcity (e.g. inequitable resource distribution and infrastructure vulnerability). This approach promotes resilient solutions that adapt to current and future needs [44].
— *Prioritize sustainable knowledge translation pathways.* Create communication tools that fit your audience, simplify complex findings for diverse audiences and actively develop long-term collaborative partnerships between actors in research, policy and practice. Identifying and investing in knowledge translation pathways that enhance the accessibility and usability of research findings helps research reach decision-makers and communities, promoting broad, sustained impact [10].
— *Align scientific research with practical and sociocultural contexts.* Engaging in diverse collaborations ensures research informed by relevant socio-economic and cultural perspectives supports sustainable and appropriate outcomes for partner communities and stakeholders [45]. Understanding the societal networks and cultural contexts that shape water-related issues and the implementation of solutions allows hydrologists to incorporate local priorities and practical constraints into their research [46].
— *Design accessible communication tools for knowledge sharing.* To bridge gaps between scientific research and local communities, hydrologists can prioritize communication tools that make complex research findings accessible and actionable [47]. Ideas include developing visualizations, simplified data summaries and community-oriented platforms that translate findings into practical terms. Tools like community workshops, digital dashboards and visual storytelling can help translate scientific knowledge into actionable insights for diverse audiences, fostering a two-way flow of information.

### (b) Incorporating diverse knowledge and co-producing research

Hydrology research must prioritize integrating diverse knowledge systems and co-producing research with those most affected by water challenges to generate effective, socially relevant solutions. Traditional hydrology often follows a unidirectional model, where scientific knowledge is created in isolation, removed from the lived experiences of communities that will ultimately implement these findings. This disconnect limits the effectiveness of research outcomes, as they may need to align with the priorities of those most affected by water-related risks, such as indigenous communities, farmers and urban planners [48]. For hydrology to foster sustainable, equitable and culturally relevant water management practices, researchers must move beyond isolated inquiry and actively incorporate local and practical knowledge into their processes.

Each framework we examine emphasizes co-production, advocating for the inclusion of local and indigenous knowledge, community experience and cross-sectoral perspectives. Embracing a range of epistemologies, from scientific models to experiential and cultural expertise, enhances the robustness and applicability of research since it promotes the development of context-specific study and (hopefully) subsequent solutions. This approach also fosters a collaborative process that advances scientific understanding and supports sustainable, community-driven solutions in water management. To put these principles into action, hydrologists can consider the following steps:

— *Develop meaningful relationships with collaborators*. Building genuine, long-term relationships with communities is fundamental to co-creation, emphasizing partnerships rooted in mutual respect, trust and reciprocal exchange [49]. This approach promotes continuous dialogue, shared responsibility and adaptability, treating collaborators as active contributors to research and outcomes. Hydrologists can develop research that benefits some of those most affected by water challenges by investing time and effort in understanding collaborators’ priorities, experiences and perspectives.
— *Integrating diverse knowledge and epistemologies.* By adopting collaborative and inclusive approaches, hydrologists can partner with local and indigenous communities to co-produce research that honours and values all knowledge systems [50]. By engaging in mutual knowledge-sharing processes, hydrologists can embrace the full context and ownership of local and indigenous knowledge [51]. These perspectives often offer unique and invaluable insights into environmental patterns, resilience strategies and regional risk factors, enriching and complementing conventional research.
— *Building inclusive, community-centred solutions in hydrology research*. Hydrologists can enhance alignment with local needs by involving diverse stakeholders—such as communities, policymakers and industry actors—throughout the research process [6]. Early engagement helps shape research questions around lived experiences, while ongoing collaboration builds trust and shared understanding. This inclusive approach supports scientifically rigorous, context-specific and culturally relevant solutions, fostering actionable pathways and meaningful, community-driven impact.
— *Develop adaptive research methodologies.* Given the complexity of water systems and their unpredictable interactions with human societies, hydrologists can adopt flexible, adaptive methodologies that respond to unexpected findings and evolving societal needs. Adaptive methods include incorporating scenario planning, iterative feedback mechanisms and adaptable research protocols that allow for adjustments based on stakeholder input and environmental changes [7] .

While the benefits of co-creation are well documented, co-production can present significant challenges, including balancing scientific rigour with practical relevance, integrating diverse knowledge systems and ensuring equitable stakeholder engagement. Differences in terminologies, worldviews and power dynamics can complicate collaboration. At the same time, institutional barriers and rigid funding structures often prioritize traditional disciplinary research over inclusive approaches that take significantly more time and funding. In addition, the complexity and uncertainty of water systems demand adaptive and iterative processes, which require time, resources and sustained effort. Building trust and long-term relationships is essential but can be hindered by historical inequities or stakeholder scepticism. Finally, evaluating the impact of co-produced research can be challenging, as success often depends on context-specific outcomes rather than conventional academic metrics. Overcoming these obstacles requires flexibility, reflexivity and a commitment to fostering meaningful partnerships and inclusive solutions.

### (c) Integrating diverse knowledge for adaptive water research

Knowledge translation in hydrology researchcan be hampered by field fragmentation because water research spans many diverse subfields [52,53]. This fragmentation risks isolated research agendas that may not fully consider the interactions between hydrological systems and societal needs. For instance, flood risk management often prioritizes engineering solutions like levees and dams, neglecting vulnerabilities and actions that exacerbate flood impacts [30]. This disconnect contributes to a gap in hydrological research outputs and the implementation of integrated water management solutions that address technical and societal challenges in an increasingly uncertain climate [54].

Fragmentation can limit the integration of diverse knowledge systems and proliferate unknowns. Effective hydrology research incorporating technical knowledge and contextual, on-the-ground insights can facilitate understanding the complex interactions between water and its surroundings [55,56]. Achieving this integration requires moving from top-down research approaches to collaborative models where community expertise, observations and lived experiences are as valued as technical data. Such inclusive methods enrich hydrology by providing context for water challenges fostering scientifically robust, culturally respectful and socially relevant solutions. Integration can bridge the gap between academic research and practical implementation to enable adaptive, context-sensitive water management. Considering hydrology through a TD lens can encourage managing disciplinary knowledge through comprehensive and adaptive research. Drawing on these frameworks, the following can help hydrologists integrate technical insights and bridge gaps between research and practical application:

— *Synthesize knowledge across subfields and sectors*. By its nature, water research spans disciplines and addressing water challenges means integrating insights from ecology, social sciences and engineering [8]. As Vogel *et al*. [57] emphasize, hydrology’s complexity demands a comprehensive approach. For instance, bringing ecological perspectives when studying river health can reveal key links between hydrological flows and biodiversity, guiding sustainable management practices. Social sciences highlight human behaviour and policy impacts essential for designing equitable water solutions, while engineering contributes the technical backbone for practical interventions. This interdisciplinary synthesis produces research that reflects the full scope of water systems, creating adaptive, resilient management strategies grounded in scientific and societal needs [39].
— *Involve community and practitioner knowledge in data interpretation*. Incorporating insights from local practitioners and communities enhances the relevance of hydrological data and improves understanding of site-specific conditions [58]. Local knowledge of historical flood patterns, drainage issues and past responses can refine models and highlight vulnerabilities often missed by technical analyses. Others have emphasized that integrating local expertise aligns research with practical realities, fostering trust and shared responsibility [59]. This collaborative approach produces robust and interoperable hydrological models, risk assessments and other outputs actionable for decision-making.

*Acknowledge research limitations and improve communication of unknowns and uncertainty.* Hydrological systems are highly complex and influenced by variables that are often difficult to quantify or predict, such as the effects of climate variability, land-use changes, and extreme weather events [60]. Unknowns can impact the accuracy of models, data interpretation and projections, and long-term impact [56]. Hydrologists should discuss their research's limitations, trade-offs, and uncertainties while considering developing audience-specific outputs tailored to the intended users. For instance, clarifying uncertainties in projections and acknowledging data gaps in climate impact studies helps policymakers and communities realistically assess associated risks. This transparency can foster informed knowledge translation so stakeholders understand the work's capabilities and constraints, leading to strong strategies.

### (d) Encourage reflexivity and adaptation

Hydrology's role in addressing society's most pressing water challenges cannot rely on rigid "business as usual" methods. Instead, it demands openness to considering diverse epistemologies that offer valuable insights, especially those outside conventional Western science norms. Reflexivity and adaptation are key to this transformation, enabling hydrologists to critically evaluate their approaches and align their work with society's complex, evolving needs. Exploring new ways to engage in research conversations, incorporating diverse and non-traditional voices, and reconsidering how knowledge is produced and shared can help bridge gaps between technical knowledge and societal needs, fostering sustainable and culturally attuned water solutions.

Central to this shift is reflexivity—critically examining how assumptions, values, biases, and positionality shape research processes, data interpretation, and conclusions. Riaux et al. [61] emphasise the need for hydrologists to confront the values embedded in their work, recognising that hydrology exists within society, not apart from it. Critically re-evaluating methodologies, tools, and frameworks enables hydrologists to question their biases and align research more directly with societal priorities. Hydrologists can ensure their work reflects diverse cultural perspectives and local contexts by embedding reflexivity and adaptability, enhancing its relevance and impact in addressing today's urgent water challenges. The following actions can support hydrologists in embedding reflexivity and adaptability into their work:

— *Critically evaluate research assumptions and processes.* Hydrologists can routinely assess the assumptions underlying their methods, tools, and techniques. Reflexive evaluation involves questioning long-held traditions about how water research can be conducted and understanding how these assumptions impact their findings and interpretations [62]. Regular peer discussions, workshops and reflexivity training can provide opportunities to recognize and address biases in research design.

— *Situate research and researcher within societal contexts*. Hydrologists should consider how their work and positionality intersect with the broader social, economic and environmental landscape, as these aspects are deeply interconnected [17,30]. Evaluating the impact of research on various communities can help ensure objectives align with the needs of those most affected by water-related issues [61]. Incorporating stakeholder feedback throughout the research process helps maintain relevance and responsiveness to societal priorities. Reflecting on positionality is equally crucial, as personal and professional identities can shape how findings are interpreted—particularly when working in contexts distinct from their lived experiences. Self-assessment tools and reflexivity exercises can help manage biases and foster an inclusive, context-aware approach to hydrological research [30].
— *Embrace adaptive research designs for uncertainty management*. Hydrologists can adopt flexible research designs to handle the inherent uncertainties in water systems. Adaptive approaches can support robust responses to changing climate impacts [54]. While considering water systems’ complexity and unpredictable interactions with human societies, hydrologists should adopt flexible, adaptive methodologies that respond to unexpected findings and evolving societal needs [56].
— *Evaluate research impact beyond traditional scientific metrics.* Hydrology research can move beyond conventional academic metrics to innovatively assess outcomes based on societal relevance, utility, scientific soundness and real-world application. Traditional metrics (i.e. h-index and citation) do not holistically reflect the broader impact of research on society, health, economy and policy. This comprehensive assessment includes gathering qualitative stakeholder feedback, tracking policy influence and measuring water systems change [63]. By doing so, hydrological research can more effectively support community resilience, inform policy and address specific water-related challenges.

## 5. Looking forward: embracing transdisciplinarity as a path forward for hydrology

Not all hydrology researchers need to shift their approach, but meaningful strides in addressing today’s significant water challenges require a willingness to go beyond ‘business as usual’ practices. For those aiming to expand their research impact, now is the time to align scientific efforts with a broader vision of societal relevance. As the global climate crisis intensifies, the demand for transformative, actionable solutions in hydrology has never been more urgent. Meeting these interconnected challenges calls for integrating science with real-world practice, engaging diverse actors and prioritizing outcomes that address societal needs.

For hydrologists exploring new approaches to address today’s urgent water challenges, the following guiding questions can aid this mindset shift:

(1) *Am I making my fundamental research accessible and open for others to build upon?* Communicating and sharing foundational insights can foster collaboration and continuous advancement, helping future researchers expand on core hydrological discoveries.
(2) *Is my applied research accessible and actionable for diverse potential end-users?* Leveraging existing and creating new pathways for making research findings relevant to stakeholders and policy can amplify the impact of applied research in addressing pressing water issues.
(3) *How can I engage communities and diverse stakeholders in both new and ongoing research to keep in alignment with urgent, real-world needs?* Engaging communities in co-production, whether in fundamental or applied research, helps align our work with the priorities and lived experiences of those affected by water challenges.
(4) *Are there alternative approaches that could better align with the goals and priorities of my research?* We can uncover new methods that drive innovative science and foster adaptive solutions by critically evaluating our tools, techniques and practices.
(5) *How can I adopt a reflexive, adaptive approach in my research?* We keep hydrological research relevant, effective and effective by regularly examining assumptions, methods and potential biases and staying responsive to societal shifts.

Practical steps can enhance the accessibility, relevance and societal impact of research. Fundamental research should be shared openly through open-access publications, user-friendly databases and workshops to enable broader collaboration and application. Applied research can become more actionable by engaging policymakers and practitioners early, translating findings into policy briefs or decision-support tools and involving end-users in its design and implementation. Engaging communities and diverse stakeholders through partnerships, inclusive communication and stakeholder mapping ensures research aligns with real-world needs and lived experiences. Exploring alternative approaches by integrating interdisciplinary methods, piloting new tools and collaborating across fields fosters innovation and adaptability. Adopting a reflexive approach through continuous feedback, self-assessment and responsiveness to societal and environmental shifts ensures research remains relevant and effective.

Hydrology must evolve to address the urgent and interconnected challenges of water management in a changing climate. The TD frameworks discussed here offer pathways for creating actionable and transformative research. Hydrologists can bridge the gap between discovery and real-world impact by integrating diverse knowledge systems, fostering deep collaboration and embedding reflexivity to deliver sustainable solutions for water systems and the communities they serve.

## Data Availability

This article has no additional data.
